# Toward an international platform: A web-based multi-language system for remote and virtual laboratories using react framework

**DOI:** 10.1016/j.heliyon.2022.e10780

**Published:** 2022-09-29

**Authors:** Zhongcheng Lei, Hong Zhou, Wenshan Hu, Guo-Ping Liu

**Affiliations:** a Department of Artificial Intelligence and Automation, Wuhan University, Wuhan 430072, China; b Center for Control Science and Technology, Southern University of Science and Technology, Shenzhen 518055, China

**Keywords:** Language scheme for internationalization, Front-end and back-end separation, Web-based online laboratories, Modular design, Engineering education

## Abstract

Universality is crucial for systems to provide services to users with different backgrounds. A good language system can remove language barriers to help to improve system universality. To improve user experience and provide new features to users, the Networked Control System Laboratory (NCSLab) has been redeveloped based on React. To achieve toward an international platform, this paper introduces a web-based multi-language system for remote and virtual laboratories based on React. The language system is implemented based on an open source tool named *react-intl-universal*. The architecture and modular design concept of the language system are investigated and the design and implementation are explored in detail from the perspective of the front-end and back-end separation scheme. The proposed language system has been integrated into the new React-based NCSLab system, which is scalable and can improve universality to serve not only domestic users in China, but also international users from all over the world.

## 1 Introduction

With the advancement of web technologies, a great many web systems have been developed in the past few decades, for example, Google, Amazon, and Microsoft. These systems provide services for different users regardless of their operating systems, web browsers, and terminal device types [1, 2]. As the world is becoming closer and closer due to the Internet, a system that provides international languages is vital to serve users throughout the world.

Among those web systems [3, 4], web-based multimedia software [5] and online learning systems such as massive open online courses (MOOCs) [6, 7] which are also called campus 2.0 [8] and web-based online laboratories [9, 10] are crucial for distance learning and online education [11, 12, 13]. Web-based online laboratories can be classified into remote laboratories, virtual laboratories and hybrid laboratories, which have been providing remote and virtual experimentation to users across the world for the last two decades. Distinguished examples are the Massachusetts Institute of Technology's iLab project for platform-independent laboratory development, scalable access and efficient management to promote online laboratory sharing [14], Stanford University iLabs initiative that turns an experiment into a data set for displaying [15], NetLab at University of South Australia for experimental circuit configuration for remote measurement [16], UNILabs at Spanish National University for Distance Education that allows controller definitions [17], VISIR at Blekinge Institute of Technology for remote wiring and measuring electronics experimentation [18], and WebLab-Deusto at University of Deusto for remote laboratory management [19].

For a general web system, the following features are considered [20, 21]:

1) *Accessibility*: The system provides web access compatible with different web browsers, terminal devices, and operating systems, and is plug-in free without crash and update issues caused by plugins such as the Flash player.
2) *Availability*: The system is available at 24/7 basis which can be accessed at any time from anywhere as long as the Internet is available.
3) *Universality:* The system provides services for different users throughout the world regardless of their languages, backgrounds, and others.
4) *Scalability*: The system supports the integration of different types of test rigs, controllers, *etc.*, and the implementation of new functionalities. A modular design would improve the scalability of a system.

With the rapid development of front-end technologies, React which is the most popular front-end JavaScript framework in the world has gained widespread acceptance and has been adopted by more and more developers. The latest version of Networked Control System Laboratory (NCSLab) which is an online laboratory adopts React to construct a new architecture to provide better user experience [13, 22].

For some web systems, they are developed for domestic users, while other systems have achieved their multi-language functionality which seeks to demonstrate international impact.

This paper is within the context of web-based remote and virtual laboratories. As the remote and virtual laboratories are laboratories as a service regardless of users’ locations, anyone could be a potential user, thus, it is necessary to develop different language systems so that anyone can use and benefit from them. Typically, for domestic users, a local language system is sufficient. With the intention of expanding, a common language for international users is needed. However, there are few systematic descriptions of the details of language system implementation in the literature.

Three feasible language internationalization schemes for React framework are *react-i18next* [23], *react-intl* [24], and *react-intl-universal* [25]. *React-intl-universal* which is built on top of *react-intl* has been selected for language internationalization in this paper.

To implement a language system, the four features shared by general web systems should be given full consideration. To achieve the preceding goals, a general and extensible multi-language system has been proposed in this paper. The multi-language system is suitable for the architecture of the new React-based NCSLab and also fits the four features of web systems.

The merits of the proposed language system are:

1) Easy for maintenance and update, for new components to be added and for the development of new functionalities. Even for the addition of a new different language, the proposed system eases the implementation.
2) The initial language can adapt to the local language according to the web browser configurations when the user first logs in to the experimentation system.
3) The proposed system can provide insights and be employed for other web-based systems.

The organization of the rest of the paper is as follows. Section 2 discusses the motivation for the construction of the language system. In Section 3, the newly developed experimentation system architecture of React-based NCSLab is presented, followed by the language system architecture demonstrated in Section 4. Section 5 provides the detailed design and implementation of the language system. Section 6 introduces a case study, which is evaluated by student responses to online surveys. The paper is enclosed with a conclusion in Section 7.

## 2 Motivation

### 2.1 Remote and virtual laboratories

Remote and virtual laboratories are important tools for research and education, covering all engineering fields including control engineering such as the controller design [26], configurable control architecture [27], hybrid control laboratories [28] and large-scale virtual control equipment [29], electric and electronics engineering such as game-based learning in a virtual laboratory [30] and a remote laboratory [31], and reusable laboratory for electronics teaching [32], and materials engineering [33]. Remote and virtual laboratories can be employed for teaching and learning, especially for experimentation and provide experimental services for users throughout the world, regardless of their physical locations. Web-based remote and virtual laboratories gradually become mainstream for less intrusiveness and more portability compared with native applications, thus, universality is important.

NCSLab is an online laboratory based on Yahoo! User Interface (YUI) framework, which has been providing both remote and virtual experiments for over 15 years [34]. In late 2017, the React framework has been adopted to replace the old YUI-based NCSLab system as the YUI is out of date and is no longer actively maintained. The functionalities such as hybrid framework and three-dimensional (3-D) interactions remain while new technologies, for example, Echarts and AntD are integrated, and a front-end and back-end separation scheme has been adopted. The previous YUI-based NCSLab system adopts a ternary operator like *<en ? English: Chinese>* for language setting. The scheme requires a massive amount of effort and is difficult for maintaining and updating as different languages and characters are mixed in all source programs, which is not scalable. Moreover, the language system of the YUI-based NCSLab system cannot automatically adapt to the browser setting. Therefore, the old language system is no longer applicable.

### 2.2 Language system

Although English is a common language, parallel language use is preferred at Nordic universities [35, 36]. Only 27,874 bachelor and masters degree programs were identified as English-taught programs outside the United Kingdom, United States, Australia and Canada in May 2021 [37]. Apart from that, the need for a multi-language platform for online learning platforms has been highlighted in [38]. Therefore, the language system is important for student learning for the following reasons: Firstly, to promote universality for global users, which enables users from different countries and regions to conduct online experiments without language barriers. Secondly, proper language setting helps users with better comprehension, for example, for some control-related terms.

## 3 Experimentation system architecture

**Figure 1:**
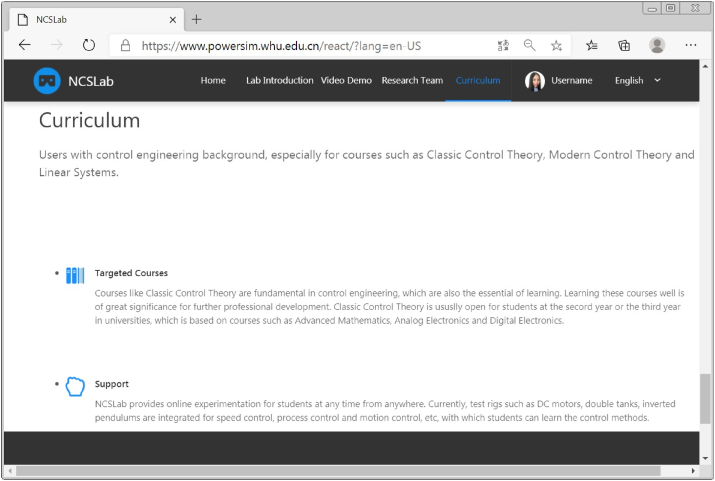
Homepage of the NCSLab experimentation system in English.

The newly developed React-based system is the NCSLab Version 5.0, which is also based on HTML5 without the installation of any plug-ins and provides 3-D interactive experimentation. The homepage of the React-based NCSLab system is shown in Figure 1, in which the portal for experimentation, laboratory introduction, video demonstration, research work, and curriculum are introduced. Currently, the system is designed mainly for university-level students with a control engineering background, and can be freely accessed by anyone through registration and email validation.

### 3.1 Four-tier architecture

**Figure 2:**
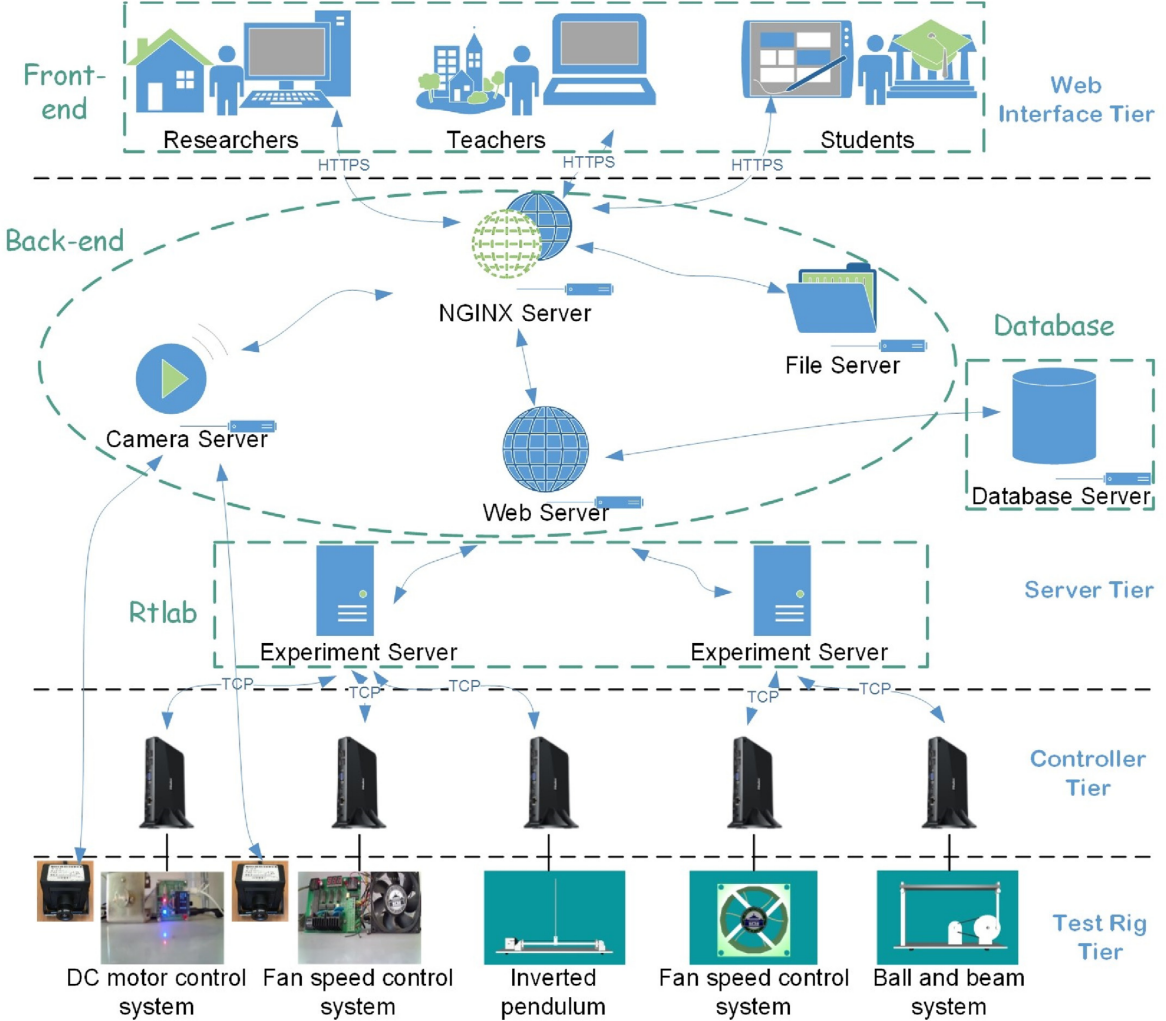
Four-tier architecture of the React-based NCSLab system.

The React-based NCSLab system can be concluded into four different tiers from the perspective of datalinks while conducting online experimentation [22], which is the same as the YUI-based NCSLab system [39, 40]. Figure 2 illustrates the four-tier architecture, which is described in detail as follows.

1) *Web Interface Tier*: Where users (researchers, teachers, and students, *etc.*) log in, monitor, and control the test rigs. Users should complete their registration process followed by activation through Email before they can log in and conduct experiments.
2) *Server Tier*: Where servers include the NGINX server, file server, database server, camera server, web server, and experiment servers. The servers are deployed at Wuhan University as a local server cluster to handle bidirectional communication between the web interface and controllers.
3) *Controller Tier*: Where control algorithms are downloaded and executed. Currently, controllers are mainly based on the Windows operating system.
4) *Test Rig Tier*: Where different physical and virtual test rigs including dc motor control systems, inverted pendulums, fan speed control systems, and ball and beam systems are integrated for remote and virtual experimentation.

From the perspective of the developer, the system can be divided into several parts as front-end, back-end, rtlab (real-time laboratory, also called the experiment server), database, and other parts concerning test rigs. The distributed architecture enables thousands of simultaneous users with configurations such as load balancing. By increasing the capacity of the servers and controllers, the system can support even more concurrent users.

### 3.2 Front-end and back-end separation scheme

**Figure 3:**
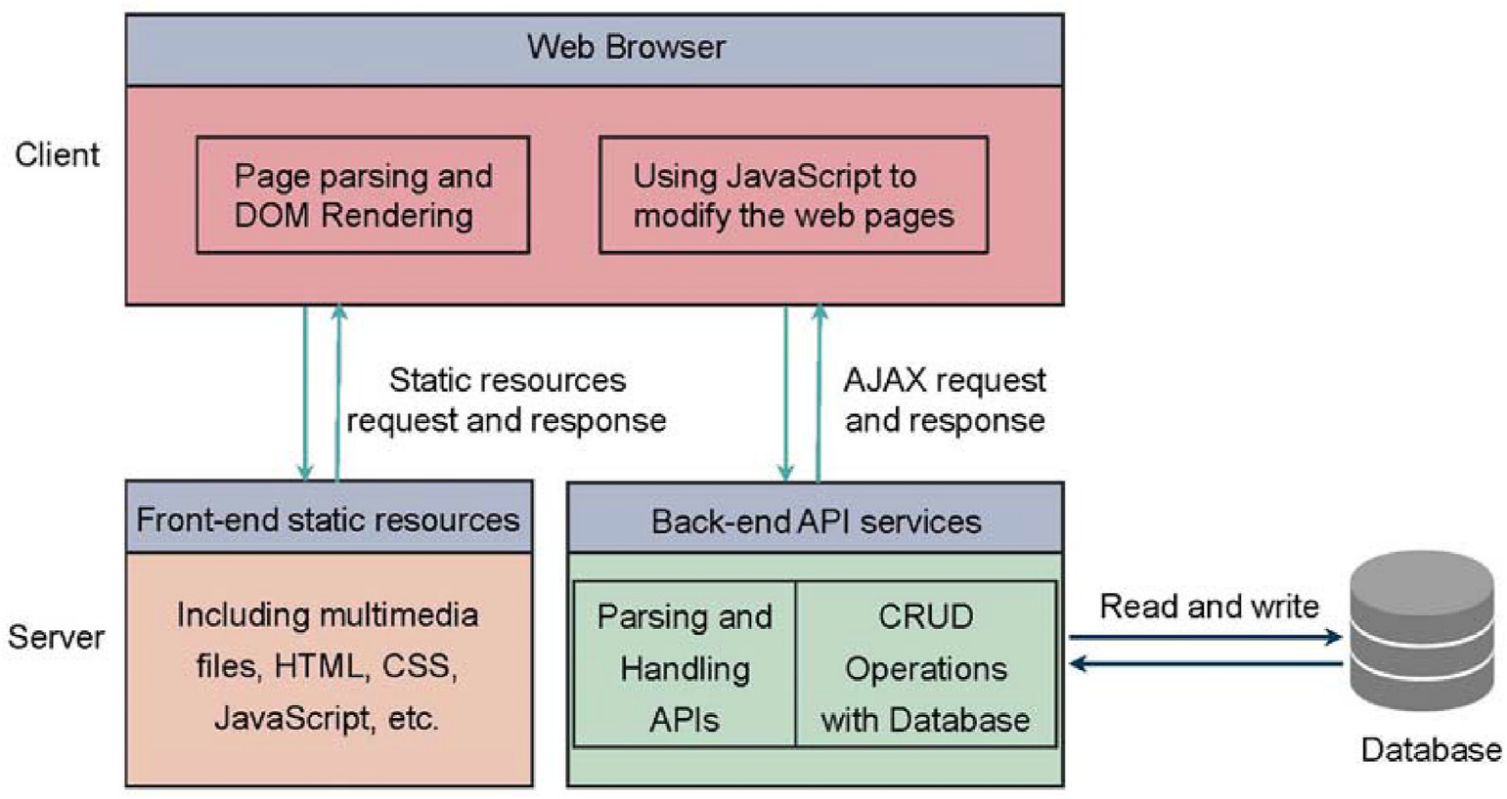
Front-end and back-end separation framework.

The React-based NCSLab system adopts a front-end and back-end separation scheme [41] to develop the whole system. Figure 3 is the front-end and back-end separation scheme adopted by the new NCSLab system [42]. Static resource requests and responses are handled by the static web server, while Asynchronous JavaScript and XML (AJAX) requests and responses are back-end services. The back-end is responsible for parsing and handling application programming interfaces (APIs), as well as CRUD (create, read, update, and delete) operations with the database.

The differences between the front-end and back-end separation framework and traditional MVC (Model, Viewer, Controller) framework are listed in Table 1. Due to the front-end and back-end separation scheme, it is easier for system development since the front-end and back-end can be separated and developed in an independent mode. However, the task for language internationalization gets harder as the data exchange mode has changed.

**Table 1:** Differences between front-end and back-end separation framework and traditional MVC framework.

Item	Front-end and back-end separation framework	Traditional MVC framework
Request static resources	A typical HTTP session	A typical HTTP session
Forward to a static page	Using front-end JavaScript to dynamically modify the web pages without communicating with back-end	Sending an HTTP request to the back-end which returns a new HTTP page
Forward to a dynamic page	Sending an AJAX request to the back-end which will interact with the database and returns data in JSON or XML format	Sending an HTTP request to the back-end which will interact with the database and returns the dynamically generated web page

### 3.3 Used technologies

Some of the technologies that are used in the design and implementation of the React-based system are closely related to the language internationalization process and are presented as follows.

1) React: React is a front-end Java Script Framework supported by Facebook, which is currently the most popular front-end framework in the world. Due to the React component (file), React provides the component modular design with great performance. In a React project, files are generally in *JSX* format which is a syntax extension to JavaScript. As the new NCSLab system is based on React, the language system should also use React.
2) Webpack: Webpack is a module bundler for bundling front-end assets such as JavaScript, HTML, CSS, and even images to improve the efficiency of web development. For Webpack, an entry file is where Webpack starts to build the bundle. Entry files can be used to load language locale files.
3) Node.js: Node.js has been installed and employed to use the command-line utility *npm* (Node Package Manager) to install Node.js libraries and applications.

## 4 Language system architecture and design concept

Supported by React, Webpack and Node.js, the language system can be constructed.

### 4.1 Architecture

Typically, a web system has one entry file, for example, the *index.html* or *index.jsp*. As an example provided by *react-intl-universal*, the entry file is *App.jsx*. However, the case is different for the new NCSLab system since the system has a complex four-tier architecture and is based on a front-end and back-end separation principle. As the new system is completely different from previous ones, the language system is supposed to be redeveloped based on the new framework.

**Figure 4:**
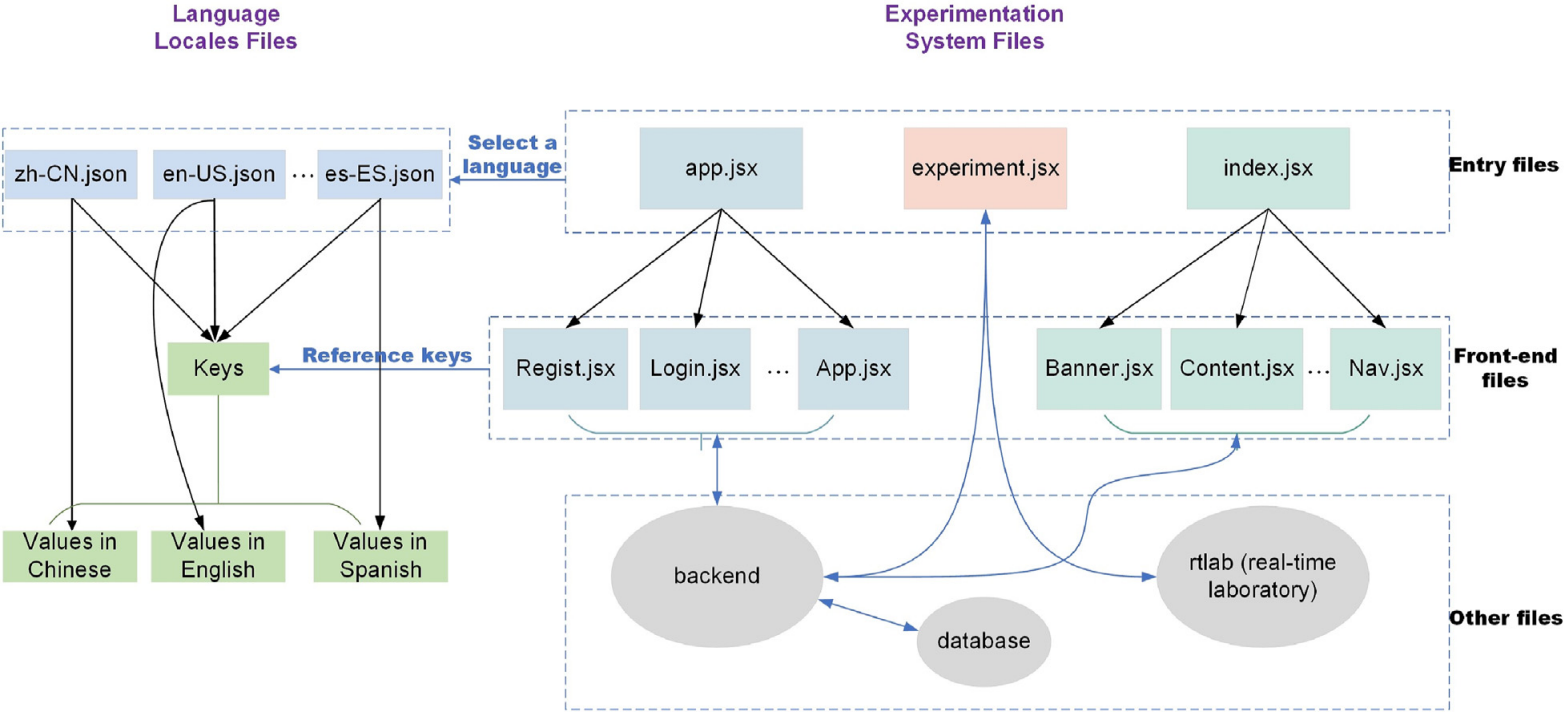
Architecture of the language system.

Figure 4 illustrates the architecture of the language system. The system source programs can be roughly classified into two categories: experimentation system files and language locale files. As defined in the four-tier architecture in Section 3.1, the experimentation system includes front-end, back-end, rtlab and database, thus, source files can be classified into frond-end files, back-end files, rtlab files, and database files.

The front-end files are composed of entry files (defined in Webpack) and other front-end files (called Front-end files in Figure 4). For the React-based NCSLab system, there are totally three entry files for experimentation system files, namely, *app.jsx*, *index.jsx* and *experiment.jsx*. The *app.jsx* is responsible for registration, login and webpages when logged in, thus, all related files are included in *app.jsx*. For *index.jsx* entry, all contents on the homepage such as the banner, navigator and footer are included. The *experiment.jsx* handles the real-time experimentation.

For language internationalization, locale files including different languages in *JSON* format are included in the entry file, thus, the entry file determines to select which language according to the corresponding configuration. By configuring entry files, other front-end files that are imported into the specific entry file can share the locale settings without further configuration. Each *JSON* file corresponds to a different language, which follows a key-value pattern. For different language configurations, the keys and corresponding values are defined. Keys are the same for different languages while values are different and are consistent with the corresponding language type. For example, in *zh-CN.json* file, the keys are the same as those in *en-US.json*, *es-ES.json*, *etc.*, while the values are in Chinese.

Keys are referenced in the front-end files. As the experimentation system adopted a complex architecture, there is a complex communication network among front-end, back-end, database, and rtlab, which also needs to be handled for language internationalization.

The official documentation provides seven types of definitions to display messages, namely, *variables*, *plural form*, *currency*, *date*, *time*, *HTML message*, and beyond that, the regular message. Table 2 shows the key-value combination examples for different types used in the React-based NCSLab system.

**Table 2:** Key-value combination examples.

Type	Key	Value in English	Configuration in JS
Regular Message	test rig status	This test rig is available	intl.get (‘test rig status')
Variable	current user	Current user: {name}	intl.get (‘current user’, {name: queueInfo.plantInfo.currentAccount})
Number	copy number	There are {plantNum, number} copies	intl.get (‘copy num’, {plantNum: plantInfo.num})
Plural Form	time remaining	{timeNum, plural, = 1 {1 min} other { # minutes}} left	intl.get (‘time remaining’,{timeNum: Math.floor (queueInfo.timeLeft/60)})

### 4.2 Modular design for future development

The proposed language system architecture adopts a modular design for future development, which enables ease of maintenance and update of the language system. The update and upgrade of the experimentation system can be concluded into the following two scenarios:

1) *Scenario 1*: Add new languages to improve universality.
2) *Scenario 2*: Add new components to integrate new test rigs or to extend functionalities.

To illustrate the modular design, the above two scenarios are considered. For Scenario 1, the following two steps are to be followed to extend the language system:

Step 1 Construct a new JSON file for a new language;

Step 2 Append a new language option to the language switch option list.

For the construction of new locale files (JSON files), only a new set of values in the form of the new language corresponding to the pre-defined keys is required, for example, the current React-based system supports Simple Chinese and English. If Spanish is to be added, only the corresponding es-ES.JSON and a switch option item are required.

For Scenario 2, the following four steps are to be followed:

Step 1 Check the corresponding entry file;

Step 2 Update JSON files to add new keys and corresponding values when needed;

Step 3 Reference keys in the front-end files where language internationalization is needed;

Step 4 Handle communication with back-end, database and rtlab when needed.

The integration of new languages, new test rigs or extension of new functionalities requires minimum effort for internationalization due to the modular design.

## 5 Design and implementation of the language system

The implementation of the language system is mainly completed in the front-end. However, due to data transmission and exchange, it also requires configuration of database, rtlab and back-end. The following subsections will discuss the enabling technologies and detailed implementation regarding database, rtlab, back-end and front-end, respectively.

### 5.1 Enabling technologies

The following packages can be installed using npm command of Node.js. If they are to be used in a React component, they need to be imported in first.

1) *react-intl-universal*: react-intl-universal is a React internationalization package developed by Alibaba Group [25], which is open source and supports more than 150 languages.
2) *qs* library: qs is a JavaScript library that can be used to parse objects. For the implementation in this paper, qs is employed to obtain the language type by parsing the URL (Uniform Resource Locator) using Location object of the Window interface which is a useful web API. Through the import of qs library, qs can be utilized to parse the URL, thus the language type is available for other configurations.

### 5.2 Internationalization implementation

Using *react-intl-universal* and *qs*, the internationalization implementation can be concluded into the following four different aspects, which have been marked in Figure 2.

**Figure 5:**
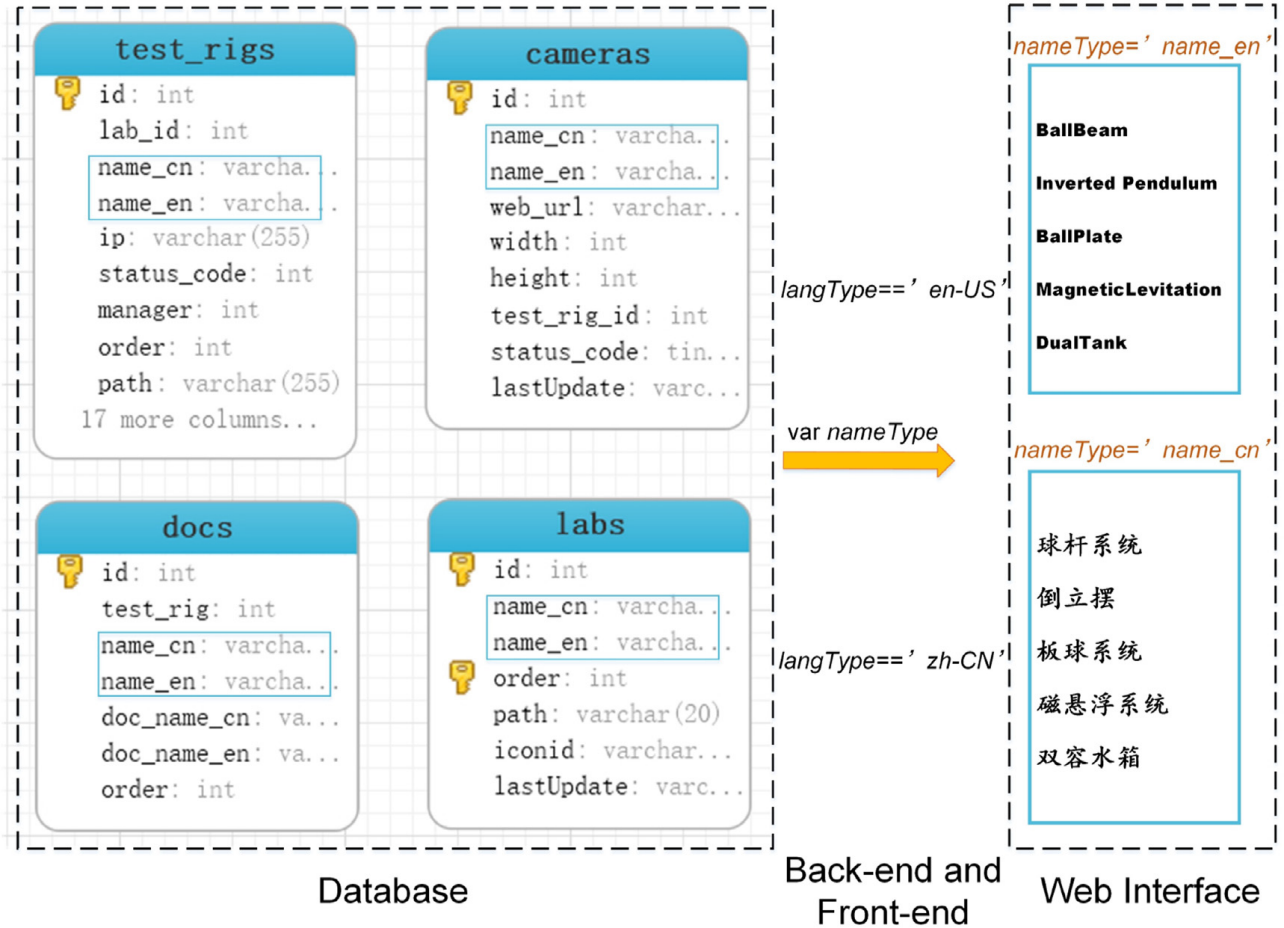
Database configuration example for internationalization.
3) ***Rtlab***: The rtlab of NCSLab system is responsible for real-time experimentation. By using the WebSocket protocol, bidirectional interaction between the rtlab and the web client is enabled [43]. Figure 6 shows the flow chart of the WebSocket scheme for the algorithm downloading and execution in the experiment server rtlab. Once a WebSocket handshake request has been received, the rtlab returns a WebSocket handshake response. Then the rtlab can actively send data to the web browser. The rtlab sends the real-time state of the algorithm using *sendText()* method, which is parsed by the front-end using *switch-case* to demonstrate different states. The internationalization is as follows: The rtlab sends the text in English, which is regarded as an expression of the *switch* statement in the front-end. All of the expression’s values are turned into case clauses, in which the message is internationalized by referencing keys. The message from rtlab, which shows the current state of the control algorithm, can not only inform the user in real time, but can also provide information for debugging if errors occur.

1) ***Back-end***: The back-end exchanges data with the front-end. For language internationalization, taking Chinese and English as examples, simply defines Chinese as *nameCN* and English as *nameEN* and references corresponding variables when necessary. Thus, when the English language is detected, *nameEN* will be referenced. Otherwise, *nameCN* will be referenced.
2) ***Database configuration***: The database structure needs to be slightly modified to allow for language internationalization. New columns of new languages are supposed to be added next to the specific field where the local language is recorded. For example, previously, the names of test rigs are recorded in Chinese, a new column in English or other languages is required for internationalization. Figure 5 illustrates a configuration example of Chinese and English. For tables *test_rigs*, *cameras*, *docs* and *labs*, a new item named *name_en* has been added and the previous name has been renamed as *name_cn*. Once the new columns for different languages are added, they can be parsed in the back-end and then referenced in the front-end. To reference the names in an appropriate language, a variable named *nameType* (represents *nameCN*, *nameEN*, etc.) has been defined, then the experience with back-end can be used in this case for language internationalization.

**Figure 6:**
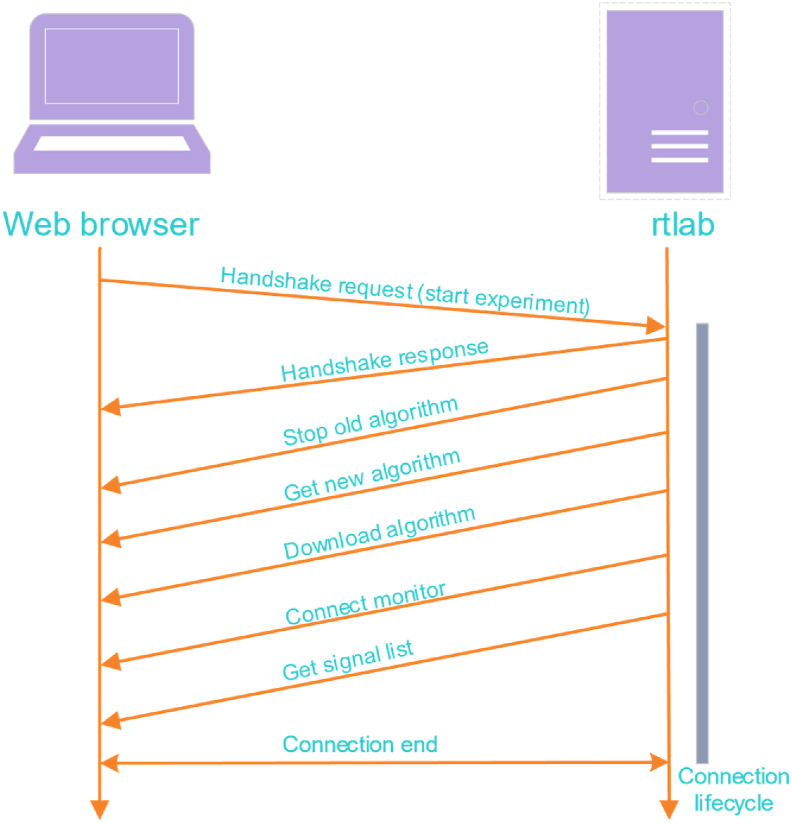
Flowchart of WebSocket scheme for algorithm downloading and execution in the experiment server rtlab.
4) ***Front-end***: The front-end handles communication with back-end and rtlab, and also the database through the back-end which works as middleware. When the back-end and rtlab are properly configured, the front-end can simply reference keys defined in *JSON* files. The *intl.get (“key”)* method is used to get the internationalized message. To reference the keys in a specific component file (a jsx file), only two steps are needed. Firstly, *react-intl-universal* should be imported into the component file. Secondly, employs the *intl.get (“key”)* method.

### 5.3 Switch option implementation

The *<select>* element is an HTML5 tag and provides a menu of options, which can be used for the control of different languages in the paper. Each language is a menu option and is defined by an *<option>* element nested inside the *<select>* element.

In order to demonstrate the currently selected language, the switch option example provided by *react-intl-universal* cannot be directly used for the proposed system. The implementation for the select switch which is used for language control in this paper is listed in line 1 to line 8 of Listing 1, in which different languages are appended into the *option* element. An event (line 10 to line 13 of Listing 1) is defined in the *<select>* element, which will be triggered once the operation of the *<select>* element is detected. The event will append the language code to the URL. The *langType* in line 3 of Listing 1 refers to the language code in the URL, which is used to be compared with the language options and ensure the desired language has always been selected, while all other language options are hidden.

**Listing 1:**
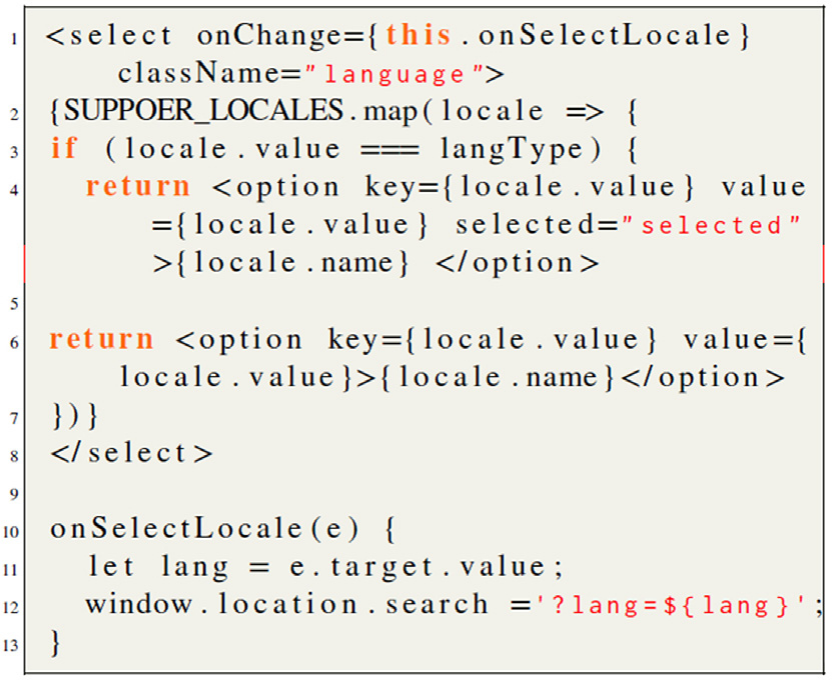
Switch option implementation.

**Figure 7:**
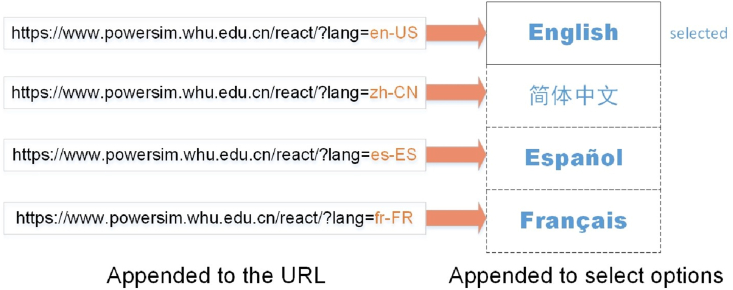
Diagram of language system implementation.

Figure 7 shows a schematic diagram of language system implementation. The selected language will appear in the select option, while other languages are hidden. Though currently only Chinese and English languages are developed, other languages such as Spanish and French can be simply integrated.

### 5.4 Language consistency within and across web pages

During the experimentation process, web pages may be refreshed and get forwarded to other pages. If not properly configured, the language may change. To keep language consistency, a *LangConsistent.jsx* component has been developed. The language of the experimentation system is consistent with the user's preferred language (usually the language of the browser UI), through the web API with *navigator.language*. The *LangConsistent.jsx* file is included in front-end files so that when users are forwarded to different webpages, the languages keep the same. Based on the React framework, Algorithm 1 shows the logic to keep language consistency within and across different pages, in which the *langType* variable has been defined. The *qs* library is used to parse the *window.location* API to get the language code in the URL, which is compared with the preferred language of the user.

**Figure:**
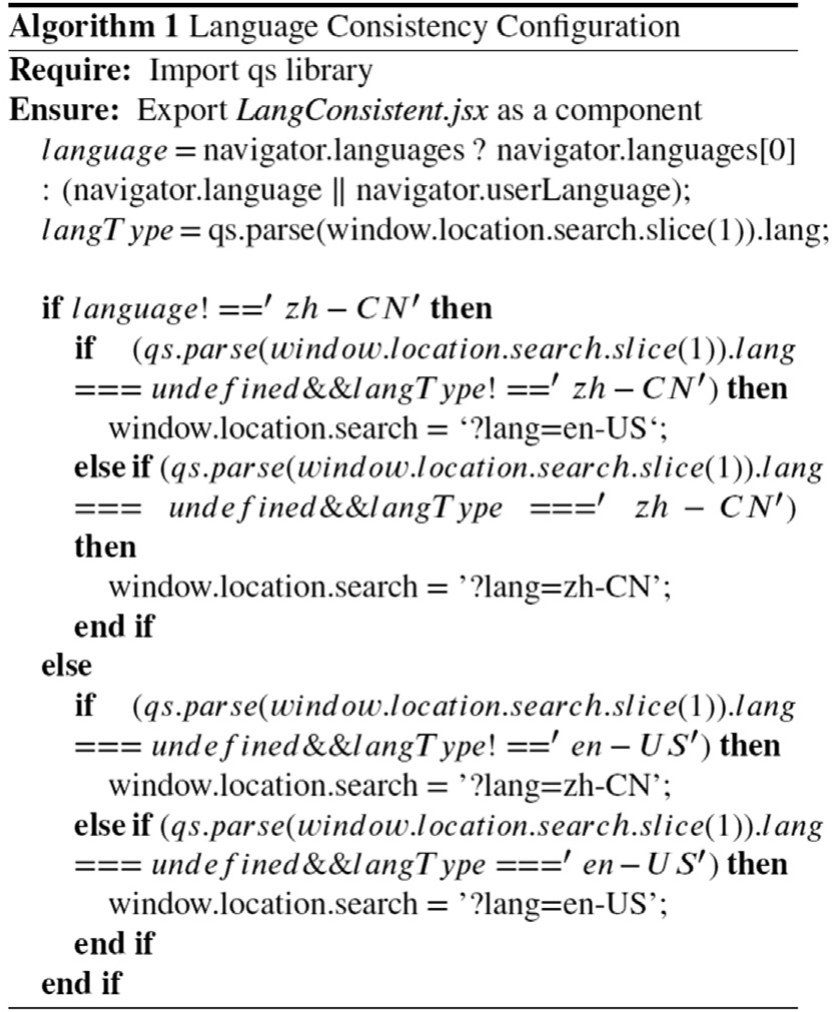

## 6 Case study

To illustrate the effectiveness of the proposed language system, the detailed language internationalization implementation of the React-based NCSLab experimentation system is provided as a case study in this section. Although currently only Chinese and English are implemented, the system is scalable and can be easily extended with new language enabled owing to the modular design.

When the proposed multi-language system has been properly deployed into the new React-based NCSLab system, users can conduct experiments with different languages. When a user first logs in, the language system can automatically adapt to the language setting of the web browser. However, if the user would like to change to a different language, he/she can switch the language by clicking the switch for a new language option.

**Figure 8:**
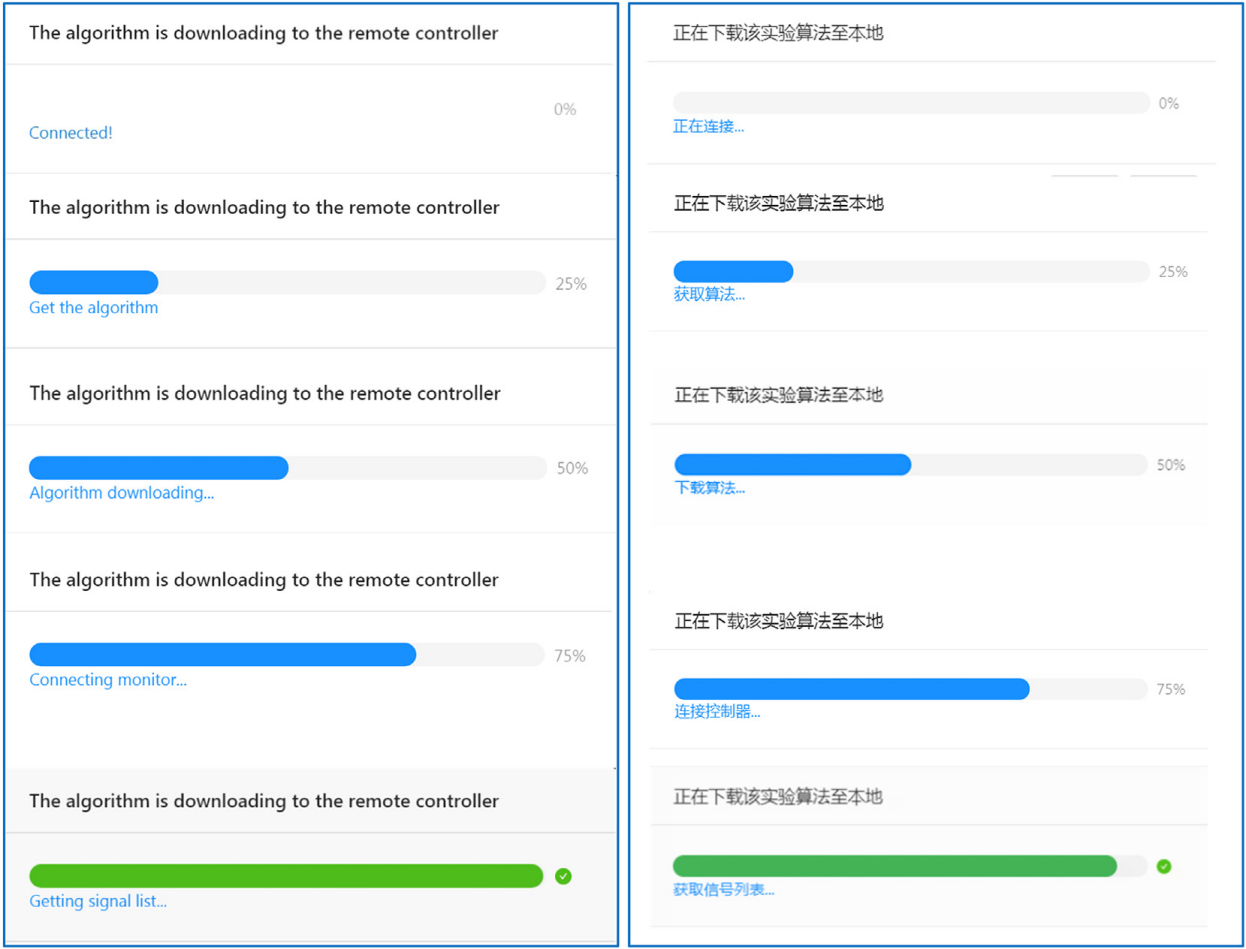
Language internationalization of algorithm downloading and execution process.

Figure 8 illustrates the language internationalization of the algorithm downloading and execution process, in which the left side is in English and the right side is in Chinese. The algorithm downloading and execution using the WebSocket protocol have been introduced in Figure 6. Figure 8 shows the processes from 0% to 25%, 50%, 75%, and 100%. If the speed of the internet connection is fast enough, the algorithm downloading process will be instant before the user notices.

**Figure 9:**
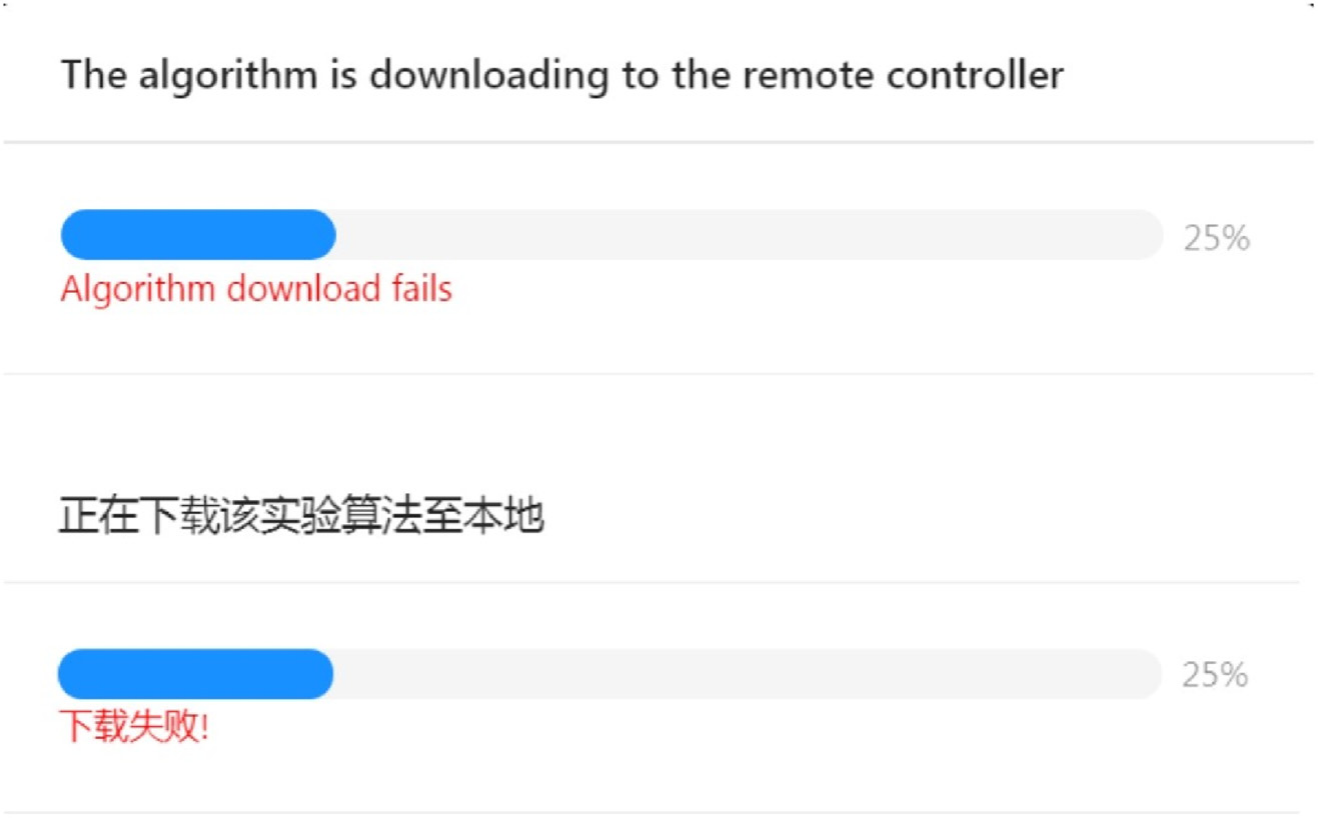
Algorithm downloading error example.

However, if an error occurs, for example, as Figure 9 (the upper of the figure is in English, and the lower is in Chinese) shows, an error occurred when the algorithm is downloaded to the remote controller at the early stage (25%), the language setting would inform the user of the error message. Then the user can ask for help, and the developer would know how to fix it. In this case, the error could be caused by the configuration of a wrong download port for the algorithm.

**Figure 10:**
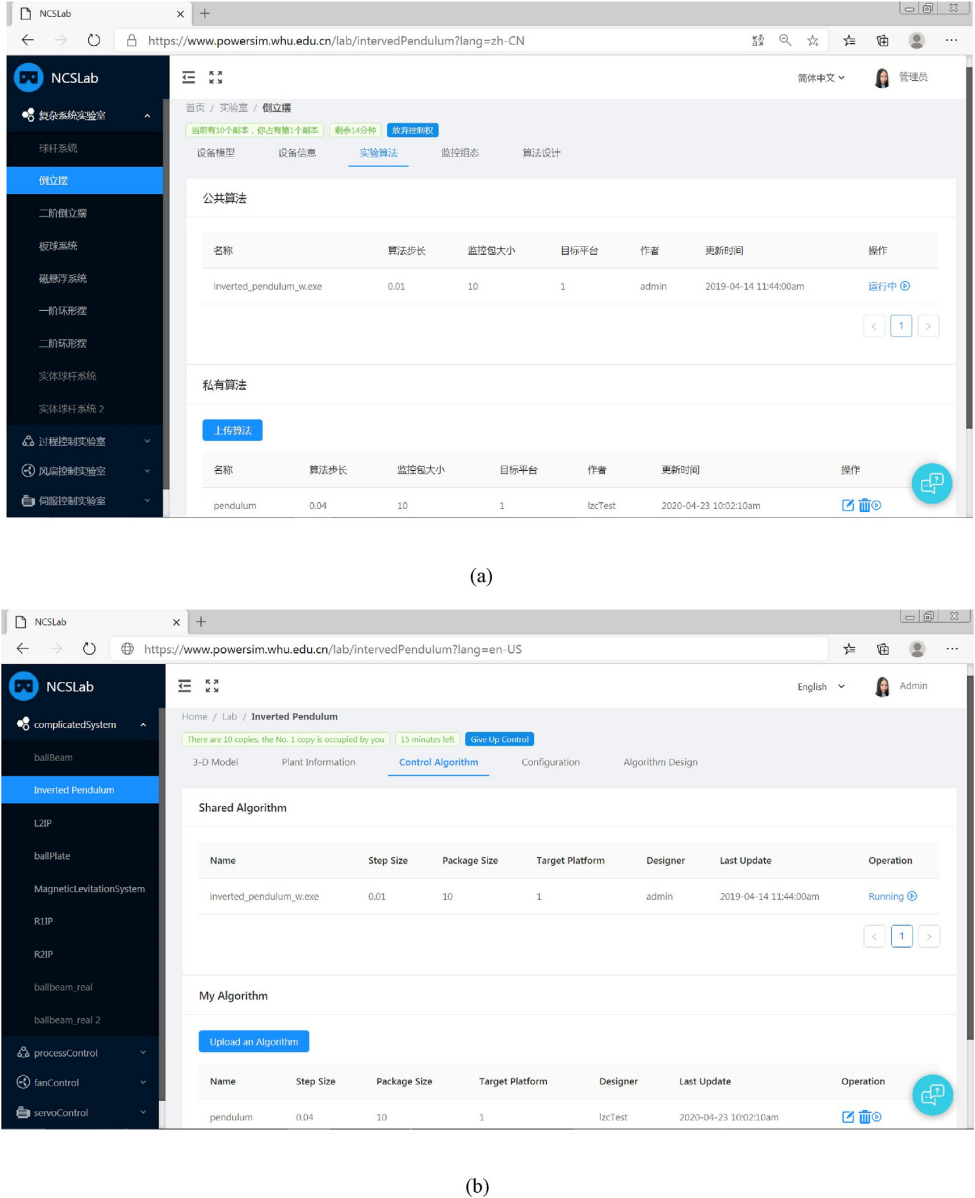
Webpage of the inverted pendulum in NCSLab. (a) In Chinese; (b) In English.

In Figure 10, the inverted pendulum is taken as an example to demonstrate the implementation result of the proposed language system, in which the control algorithm interface has been presented. The left side of Figure 10 (both Fig. 10(a) and Fig. 10(b)) is the control panel of test rigs which are cataloged into different sub-laboratories by their functionalities. On the right side of Figure 10, the details of the inverted pendulum including the 3-D model, plant information, control algorithm, configuration, and algorithm design are introduced. It can be seen that users can upload their control algorithms through the web link, and they can also share their algorithms with other users. By clicking the operation button, a WebSocket communication will be established between the web interface and the rtlab. Then the control algorithm can be downloaded to the remote controller where algorithms are executed.

**Figure 11:**
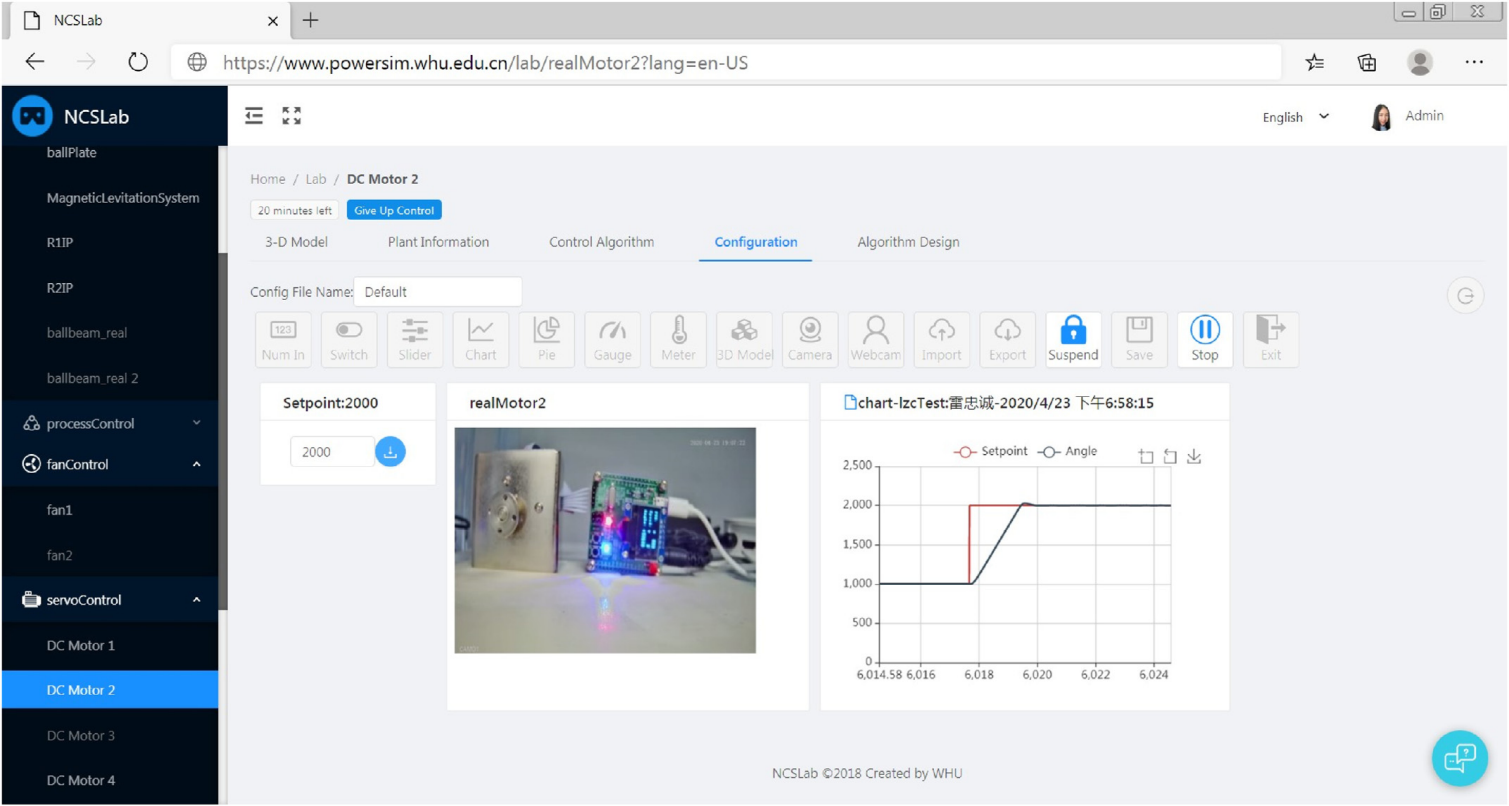
Experimentation with a dc motor control system of the NCSLab system.

Figure 11 shows an example of the real-time experimentation with a dc motor control system of the React-based NCSLab system, the interface of which is in English. It can be seen that the provided widgets and parameter names are translated into English. Both domestic users in China and international users throughout the world can benefit from the proposed language system.

Great importance has been attached to the user experience of the NCSLab system. Previous studies of NCSLab have proved the usability, educational value, and stability of the system [12, 22, 39]. The users’ attitude toward the language system is also taken into account during the design and application of the system. After the experimentation with NCSLab, an online survey was distributed to the students, and they were invited to respond to the survey. Regarding the language system, an open question “Have you encountered any language issues during your experimentation? For example, there were bugs in the Chinese interface or the English interface. If yes, please point it out for our improvement!” was provided. Table 3 shows the student responses to the online questionnaires about the language system. The data are collected from students with different backgrounds in different years. The response rates for 2020, 2021 and 2022 are 71/71, 75/75, 154/380, respectively. It can be seen from Table 3, that most students in different years responded that there were no language issues excluding the irrelevant comments. Some reported issues related to the language system are also included. It can be concluded that most students recognized the effectiveness of the language system. The main reason that there are English characters in the Chinese version is that variables and blocks in the algorithm design interface are named in English to avoid errors during algorithm generation and execution due to the Chinese character encoding issue. The garbled characters and the automatically switching were not encountered before and they need further research.

**Table 3:** Student responses to the online questionnaires on the language system.

Year	Major & University	No issue rate	Reported issues
2020	Automation at Wuhan University	92.96%	There are English characters in the algorithm design interface in the Chinese version
2021	Electronics and Information Science at Henan Agricultural University	89.33%	Some sections are garbled
2022	Electrical Engineering at Wuhan University	79.22%	Automatically switching between the English and the Chinese version

## 7 Conclusion

In this paper, a web-based multi-language system for remote and virtual experimentation systems based on React framework was discussed. The aim of the paper is to provide a web-based language system to achieve toward an international platform. The language system architecture and modular design are provided, which enables ease of maintenance and update with new test rigs and new functionalities integrated. Moreover, even for the addition of a new different language, the proposed system can ease the implementation, which would cost a great amount of effort using traditional methods. The proposed system can remove language barriers and is of great scalability and provides universality for online experimentation. The system has been proven helpful and useful during the intensive construction of the new experimentation system in the past few years. The proposed language system can be potentially applied to other systems owing to its simplicity and effectiveness. Currently, only a few international users used the NCSLab, and their experience with the language system was not collected yet. The feedback from international users should be considered in the future.

## Declarations

### Author contribution statement

Zhongcheng Lei: Conceived and designed the experiments; Performed the experiments; Analyzed and interpreted the data; Contributed reagents, materials, analysis tools or data; Wrote the paper.

Hong Zhou: Contributed reagents, materials, analysis tools or data.

Wenshan Hu: Analyzed and interpreted the data; Contributed reagents, materials, analysis tools or data; Wrote the paper.

Guo-Ping Liu: Analyzed and interpreted the data; Contributed reagents, materials, analysis tools or data.

### Funding statement

This work supported by National Natural Science Foundation of China [62103308; 62073247; 62173255; 62188101], China Postdoctoral Science Foundation [2022T150496].

### Data availability statement

No data was used for the research described in the article.

### Declaration of interest's statement

The authors declare no conflict of interest.

### Additional information

No additional information is available for this paper.
